# Atorvastatin Improves Inflammatory Response in Atherosclerosis by Upregulating the Expression of GARP

**DOI:** 10.1155/2015/841472

**Published:** 2015-05-10

**Authors:** Xiaoqi Zhao, Yuzhou Liu, Yucheng Zhong, Bo Liu, Kunwu Yu, Huairui Shi, Ruirui Zhu, Kai Meng, Wei Zhang, Bangwei Wu, Qiutang Zeng

**Affiliations:** Department of Cardiology, Union Hospital, Tongji Medical College, Huazhong University of Science and Technology, Wuhan 430022, China

## Abstract

Regulatory T cells play an important role in the progression of atherosclerosis. GARP is a newly biological membrane molecule existed on activated Tregs, which is related to the release of TGF-*β*. The antiatherosclerosis effects of statins partly depend on their multiple immune modulatory potencies. In this paper, we present that atorvastatin could upregulate the expression of GARP and TGF-*β* in CD4+ T cells and increase the numbers of CD4+LAP+ and CD4+Foxp3+ regulatory T cells in ApoE−/− mice. Also, we indicate that atorvastatin promotes the aggregation of GARP+ and Foxp3+ cells and secretory of the TGF-*β*1 in atherosclerotic plaques. Furthermore, we prove that atorvastatin could delay the procession of atherosclerosis and improve the stability of atherosclerotic plaques. Interestingly, we report that inhibition of GARP distinctly inhibits the anti-inflammatory effects of atorvastatin. We conclude that atorvastatin improves the inflammatory response in atherosclerosis partly by upregulating the expression of GARP on regulatory T cells.

## 1. Introduction

Atherosclerosis is regarded as one kind of immune-inflammatory disease characterized by accumulation of lipids in the artery wall and progressive arterial stenosis and ischemic symptoms [1]. Coronary atherosclerotic heart disease manifests as progressive lipid infiltration and narrowing of the coronary artery, which remains a leading cause of death and loss of productive life years in the world with consequences such as myocardial infarction [2]. The activation of vascular endothelial cells generates the aggregation of inflammatory cells into the atherosclerotic lesion [3]. The macrophages, derived from the differentiation of monocytes by secretory proinflammatory cytokines, further turn into foam cells by uptake of oxLDL and sequentially enhance the inflammatory response, thereby recruiting much more immune cells [4]. In addition, solid evidence has indicated that T helper type 1 (TH1) and T helper type 17 (TH17) cells-mediated immune responses effectively promote the progress of atherosclerosis [5].

Regulatory T cells (Tregs) are the naturally (nTregs) or inductively (iTregs) produced key suppressors in self-reactive immune responses [6]. Tregs can slower development of atherosclerosis by inhibiting immune overactivity of effector T cells and secrete major anti-inflammatory cytokines, such as interleukin-10 (IL-10) and transforming growth factor (TGF-*β*), which are able to reduce inflammatory response in atherosclerosis [7]. Both the animal and clinical trials have demonstrated that the quantity and anti-inflammatory functions of Tregs are impaired in atherosclerotic individuals [8].

The 3-hydroxy-3-methylglutaryl coenzyme A (HMG-CoA) reductase inhibitors, also named statins, are originally designed to reduce blood lipid. Recently, increasing evidence has recognized statins as potential anti-inflammatory agents [9]. Statins have extensive effects on both the innate and the adaptive immune systems and acting on many kinds of immune cells, including macrophages, dendritic cells, and T cells [10]. Growing evidences have demonstrated that statins could induce the accumulation of Tregs in atherosclerotic plaque in mouse model and improved the quantity and anti-inflammatory functions of Tregs in ACS patients [11–13].

Glycoprotein A Repetitions Predominant (GARP), also known as Leucine Rich Repeat Containing 32 (LRRC32), is an orphan toll-like receptor composed of leucine-rich repeats, which has been regarded as a potential surface marker of activated Tregs [14, 15]. TCR stimulation (such as anti-CD3/CD28) is required for efficient anchoring of GARP to the Treg surface [16, 17]. The efficient anchoring of GARP into Tregs surface appears to mediate upregulation of Foxp3, which strengthens Tregs to suppress the activation of effector T cells [16–18]. Recently, it has been reported that GARP could bind directly to TGF-*β* latency-associated protein (LAP), which is expressed on the surface of nTreg and the upregulation of LAP on activated Tregs correlated with GARP upregulation [19–22]. However, it has been reported that Foxp3 is not necessary for the expression of GARP and LAP on human Tregs, because the expression of GARP and LAP is normal following knockdown of Foxp3 by siRNA [23]. More interesting, some researchers have described a new population of regulatory T cells expressing LAP; these CD4+LAP+ T cells lack Foxp3 but secrete IL-8, IL-9, IL-10, IFN-*γ*, and TGF-*β* upon activation, and they exhibit immune-suppressive activity in vitro [24]. Furthermore, other researchers propose that the latent TGF-*β* anchoring in T cell surface mediated by binding to GARP may be required for the effect of Tregs on activation of TGF-*β* after TCR stimulation [17, 25]. Generally speaking, the activity of latent TGF-*β* and Foxp3 anchoring in Tregs is closely related to the presence of GARP.

Due to the pleiotropic effects of statins on regulation of inflammation, firstly, we explored the influence of atorvastatin on mouse ant-CD3/CD28 stimulated splenic CD4+ T cells in vitro. Interestingly, an increased expression of GARP and TGF-*β* was observed in this in vitro experiment. In vivo, after the administration of oral atorvastatin we observed an upregulation of CD4+LAP+ and CD4+Foxp3+ regulatory T cells in ant-CD3/CD28 stimulated ApoE−/− splenic cells. At the same time, we recruited the lentivirus-GARP-siRNA to inhibit the expression of GARP, in order to reveal the core effects of GARP in this phenomenon. We also detected the recruitment of GARP+ and Foxp3+ cells, as well as the TGF-*β* secretory in atherosclerotic plaques after the administration of oral atorvastatin or simultaneously lentivirus-GARP-siRNA intervention. At last, we observed the immune-modulatory effects of atorvastatin and lentivirus-GARP-siRNA on progress of mouse atherosclerosis. Conclusion, we firstly propose that atorvastatin regulates inflammatory reaction in atherosclerosis at least partly through upregulating the expression of GARP.

## 2. Methods and Materials

The institutional ethics committee of Huazhong University of Science and Technology approved the animal use in this study.

### 2.1. Atherosclerosis Mice Model

Eight-week-old male ApoE−/− mice (C57BL/6 background) were continuously fed with a high-fat Western diet (0.25% cholesterol and 15% cocoa butter) and housed under standard conditions (12 h light/12 h dark cycle, 22~25°C room temperature). The mice were randomly divided into three groups, the oral atorvastatin treatment group (O.A. + scrambled, oral atorvastatin 80 mg/Kg/day in 150 *μ*L PBS + 5 × 10^7^ lentivirus-negative control scrambled sequence intravenous injected per week), oral atorvastatin treatment accompanied by lentivirus-siRNA mediated knockdown of GARP group (O.A. + GARP-siRNA, oral atorvastatin 80 mg/Kg/day in 150 *μ*L PBS + 5 × 10^7^ lentivirus-GARP-siRNA intravenous injected per week), and scrambled group (scrambled, oral 150 *μ*L PBS per day + 5 × 10^7^ lentivirus-negative control scrambled sequence intravenous injected per week); each group had 10 mice (*n* = 10). The siRNA lentiviral vector against GARP (LV-LRRC32-RNAi; 14644-2) and negative control scrambled sequence (-TTCTCCGAACGTGTCACGT-) were designed and synthesized by GENECHEM Co., Ltd., Shanghai, China. Four weeks later, mice in these three groups were euthanized, and specimens (including peripheral blood, spleen, heart, and aorta) of them were collected for further research.

### 2.2. Atherosclerotic Lesion Analysis

The heart and aorta of these mice were washed with PBS and stained by 4% paraformaldehyde for 2 hours. The hearts were half cut and the upper parts were embedded in OCT compound, sliced in 8 *μ*m thickness, mounted on slides, and stained with oil red O. For analysis of atherosclerotic lipid plaques, a section in the middle of the three aortic valves was used, and insuring visible of the valves and their attachment sites on these sections. Masson trichrome staining was achieved with Masson Trichrome Stain Kit (Sigma-Aldrich Inc., St. Louis, MO, USA). Lesion areas were measured from the intima to the lumen. The areas were measured by Image Pro plus 6.0 (public domain software).

En face lipid plaques of the entire aorta were also analyzed. After flushing and fixation, the whole aorta was dissected out from ascending aorta and its bifurcation to bilateral common iliac arteries, nailed on a black wax pan and stained with oil red O. The total areas and the atherosclerotic lesions of each aorta were also measured by Image Pro plus 6.0.

### 2.3. Cell Isolation and In Vitro Culture

The splenic cells were obtained by milling the spleen and filtering the cells through a 40 mm filter. Lymphocytes were isolated from total splenic cells by density-gradient centrifugation using Lymphocyte separation medium (MP Biomedicals Co., Ltd., USA). Splenic lymphocytes were cultured in 24-well plates (stimulated by plate-bound 5 *μ*g/mL anti-CD3/CD28, 1 × 10^7^ cells/mL) at 37°C in an atmosphere of 5% CO_2_ in RPMI 1640 medium (HYCLONE, Co., Ltd., USA) supplemented with 10% fetal bovine serum (Gibco-BRL, Grand Island, NY, USA), 1% penicillin-streptomycin, and 1% glutamine. 25 *μ*M atorvastatin (Sigma-Aldrich Inc., St. Louis, MO, USA) was added into stimulated splenic lymphocytes and incubated for 96 hours. The knockdown of GARP in vitro was achieved by lentivirus-GARP-siRNA (MOI = 100). The cells cultured with scrambled lentivirus (MOI = 100) were set as control. For cell sorting, CD4+ T cells were isolated from splenic lymphocytes with a negative CD4+ T cell isolation kit (Miltenyi Biotec, Bergisch Gladbach, Germany), and Tregs were isolated from splenic lymphocytes by using CD4+CD25+ Regulatory T Cell Isolation Kit (Miltenyi Biotec, Bergisch Gladbach, Germany) and cultured under the appropriate conditions (5 *μ*g/mL anti-CD3/CD28 and 10 *μ*g/mL IL-2).

### 2.4. Flow Cytometry

Lymphocytes were isolated from mouse spleen by density gradient centrifugation. After 24 h activation by plate-bound 5 *μ*g/mL anti-CD3/CD28, lymphocytes were harvested for flow cytometry to assess the proportion of CD4+LAP+ and CD4+Foxp3+ regulatory T cells. Prepared lymphocytes were incubated at 4°C in the dark for 30 mins with fluorescein isothiocyanate- (FITC-) labeled anti-mouse CD4 antibodies (eBioscience), PerCP-Cy5-5- (Cy5-5-) labeled anti-mouse LAP antibodies (eBioscience), and allophycocyanin- (APC-) labeled anti-mouse GARP antibodies (eBioscience) for surface staining. To measure intracellular staining of Foxp3, cells were washed with PBS after surface staining for CD4 and GARP, fixed in 4% paraformaldehyde solution and then permeabilized. Permeabilized cells were stained with fluorescent PE-labeled anti-mouse Foxp3 antibody (eBioscience), diluted in permeabilization buffer for 1 hour. For the detection of Th1, Th2, and Th17, lymphocytes were stimulated by PMA (100 ng/mL)/lonomycin (750 ng/mL) for 4 hours, washed with PBS after surface staining for FITC-CD4, fixed in 4% paraformaldehyde solution, and then permeabilized; permeabilized cells were stained with fluorescent PE-labeled anti-mouse INF*γ*, IL-4, or IL-17 antibodies (eBioscience) diluted in permeabilization buffer for 30 mins. At last, these cells were washed for 2~3 times and analyzed by a BD FACScan. The results of flow cytometry were analyzed by Flowjo 7.6.3.

### 2.5. Apoptosis Analysis in Tregs

MouseTregs were isolated from the fresh splenic lymphocytes by using CD4+CD25+ Regulatory T Cell Isolation Kit (Miltenyi Biotec, Bergisch Gladbach, Germany) and then cocultured with scrambled siRNA (MOI = 100) or GARP-siRNA (MOI = 100) under the appropriate conditions (5 *μ*g/mL anti-CD3/CD28 and 10 *μ*g/mL IL-2) for 24 hours, and fresh isolated Tregs were cultured under the appropriate conditions for 24 hours as control. Apoptotic cells were identified by staining for annexin V (Annexin V-APC Apoptosis Detection Kit, Biolegend). For Tregs apoptosis analysis, Tregs were suspended in 100 *μ*L annexin V binding buffer (Biolegend), after incubation with 5 *μ*L APC-annexin V for 15 min at room temperature; these cells were resuspended in 400 *μ*L annexin V binding buffer and analyzed by a BD FACScan. The results of flow cytometry were analyzed by Flowjo 7.6.3.

### 2.6. Tregs Functional Suppression Assays

Mouse Tregs (2 × 10^5^) sorted from different groups (scrambled group, lymphocytes treated by scrambled siRNA with MOI = 100 in vitro for 96 h; atorvastatin group, lymphocytes treated by 25 *μ*M atorvastatin + scrambled siRNA with MOI = 100 in vitro for 96 h; atorvastatin + GARP inhibition group, lymphocytes treated by 25 *μ*M atorvastatin + GARP-siRNA with MOI = 100 in vitro for 96 h) were cocultured with syngeneic ApoE−/− mouse CD4+CD25− effector T cells at different suppressor-responder ratios (1 : 4, 1 : 2, and 1 : 1) in a 96-well plate supplemented with 5 *μ*g/mL anti-CD3/CD28 and 10 *μ*g/mL IL-2, in the presence of CD4− cells pretreated with mitomycin C (Sigma-Aldrich, St. Louis, MO, USA). All groups were in triplicate. After 72 hours, [^3^H] thymidine (1 *μ*Ci/well) was added for 16 hours before scintillation counting with a beta counter. Percentage inhibition of proliferation was determined by the following formula: 1-(median [^3^H] thymidine uptake of Tregs: CD4+CD25− coculture/median [^3^H] thymidine uptake of CD4+CD25− cells).

### 2.7. RT-PCR

The total RNAs were isolated from mouse samples using RNAiso Plus (TakaRa, Japan) following the manufacturer's protocol. Equal quantity of the mRNAs was synthesized for cDNA by using a PrimeScript RT reagent kit (TakaRa, Japan). The specific primers for RT-PCR were synthesized by Newtsingke (Peking, China). The primer sequences were as follows: GARP forward 5′-TGAATTCATGAGCCACCAGATCCTGCTACTC-3′ and reverse 5′-AGCGGCCGCTCAGGCTTTGTATTGTTGGCTGAG-3′; TGF-*β* forward 5′-GTGTGGAGCAACATGTGGAACTCTA-3′ and reverse 5′-TTGGTTCAGCCACTGCCGTA-3′; IL-10 forward 5′-GCTCTTACTGACTGGCATGAG-3′ and reverse 5′-CGCAGCTCTAGGAGCATGTG-3′; GAPDH forward 5′-GCACAGTCAAGGCCGAGAAT-3′ and reverse 5′-GCCTTCTCCATGGTGGTGAA-3′. The expression of these mRNAs was quantified by real-time polymerase chain reaction (SYBR Green Master Mix, Takara, Japan) with an ABI PRISM 7900 Sequence Detector system (AB Applied Biosystems). The cycling program included 20-second initial preincubation at 95°C followed by 35 cycles of 95°C for 10 seconds, 60°C for 20 seconds, and 70°C for 1 second.

### 2.8. Western Blot

The protein was collected from aortas and anti-CD3/CD28 stimulated splenic CD4+ T cells from each group using lysing buffer (PBS with 1% Nonidet P-40, 0.5% sodium deoxycholate, 0.1% SDS, 0.1 mg/mL phenylmethyl sulfonyl fluoride, 0.3 TIU/mL aprotinin) on ice for 30 mins. Cells or tissue homogenates were then centrifuged at 12,000 g for 15 min at 4°C. Protein concentration was measured in the supernatants using the BCA Protein Assay Kit (Beyotime Institute of Biotechnology). 20 *μ*g of protein was separated by SDS-PAGE and transferred into polyvinylidene fluoride microporous membranes (Bio-Rad Laboratories, Hercules, CA). The membrane was blocked with 5% milk in 0.5% Tris-buffer saline solution (pH 7.6) for 1 hour and then incubated with primary antibodies for GARP (Aviva Systems Biology), TGF-*β* (Santa Cruz's Biotechnology, Inc., for precursor and mature TGF-*β*1), Smad3/p-Smad3 (Santa Cruz's Biotechnology, Inc.), and GAPDH (EPITOMICS) overnight at 4°C. The membrane was then incubated for 30 mins with HRP conjugated secondary antibody (KPL Co., Ltd.) at room temperature. The blots were visualized using ECL Western Blotting Detection Reagents (Bio-Rad Laboratories, Hercules, CA).

### 2.9. Immunohistochemistry

The middle aortic valves of each mice were separated and the frozen consecutive sections of 5 *μ*m thickness were collected for immunohistochemical assay, including GARP (1 : 200; Aviva Systems Biology, USA), mature TGF-*β* (1 : 100; Santa Cruz's Biotechnology, Inc., for precursor and mature TGF-*β*1), Foxp3 (1 : 200; eBioscience, USA), CD3 (1 : 200; Abcam, UK), and CD68 (1 : 200; Fitzgerald, USA). Image Pro Plus 6.0 software were used to analyze the results, and the analysis was performed by an observer blinded to this research.

### 2.10. Statistics

All values are expressed as means ± SEM, and *P* values <0.05 were regarded as significant. We performed analysis of significance in Prism (GraphPad 6.0) by the Mann-Whitney test.

## 3. Results

### 3.1. Atorvastatin Upregulated the Expression of GARP and TGF-*β* in Anti-CD3/CD28 Stimulated Splenic CD4+ T Cells

**Figure 1:**
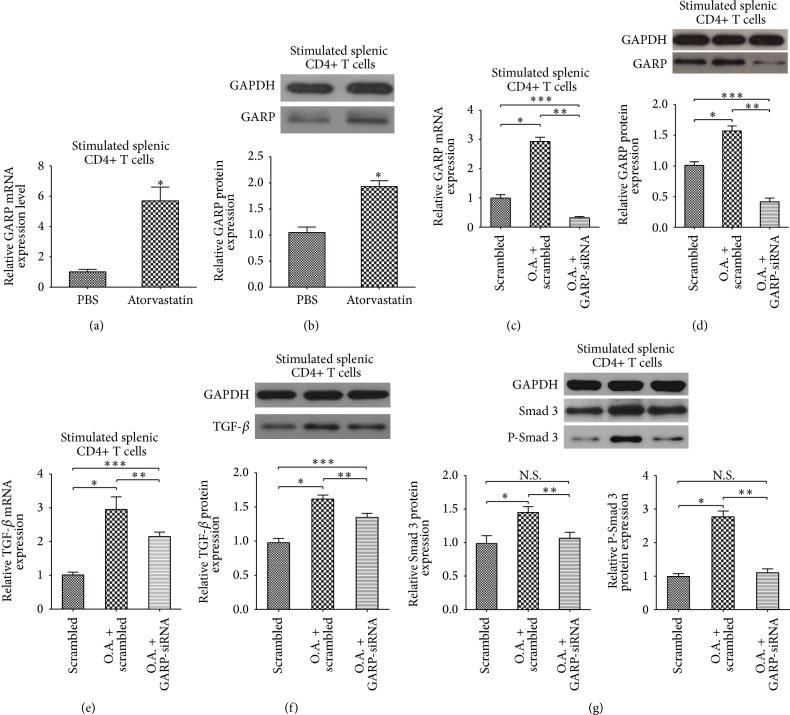
Atorvastatin upregulated the expression of GARP in anti-CD3/CD28 stimulated splenic CD4+ T cells: CD4+ T cells were isolated by magnetic activated cell sorting (MACS). (a) The relative mRNA expression of GARP in atorvastatin cocultured stimulated splenic CD4+ T cells, in vitro. Repeated times *n* = 6, and ^∗^ *P* < 0.01 compared with the control wells. (b) The relative protein expression of GARP in atorvastatin cocultured stimulated splenic CD4+ T cells, in vitro. Repeated times *n* = 6, and ^∗^ *P* < 0.05 compared with the control wells. (c) The relative mRNA expression of GARP in stimulated splenic CD4+ T cells derived from mice in scrambled group (scrambled), oral atorvastatin + scrambled group (O.A. + scrambled), and oral atorvastatin + GARP-siRNA group (O.A. + GARP-siRNA), in vivo. Data were from 6 mice per group (*n* = 6) and were independently confirmed, all ^∗^ *P*, ^∗∗^ *P*, and ^∗∗∗^ *P* < 0.05. (d) The relative protein expression of GARP in stimulated splenic CD4+ T cells derived from mice in these three groups, in vivo. Data were from 6 mice per group (*n* = 6) and were independently confirmed, all ^∗^ *P*, ^∗∗^ *P*, and ^∗∗∗^ *P* < 0.05. (e) The relative mRNA expression of TGF-*β* in stimulated splenic CD4+ T cells derived from mice in these three groups, in vivo. Data were from 6 mice per group (*n* = 6) and were independently confirmed, all ^∗^ *P*, ^∗∗^ *P*, and ^∗∗∗^ *P* < 0.05. (f) The protein expression of TGF-*β* in stimulated splenic CD4+ T cells derived from these three groups, in vivo. Data were from 6 mice per group (*n* = 6) and were independently confirmed, all ^∗^ *P*, ^∗∗^ *P*, and ^∗∗∗^ *P* < 0.05. (g) The relative protein expression of total Smad3 and phosphorylated Smad3 (P-Samd3) in stimulated splenic CD4+ T cells derived from mice in these three groups, in vivo. Data were from 6 mice per group (*n* = 6) and were independently confirmed, all ^∗^ *P* and ^∗∗^ *P* < 0.05.

In order to identify the in vitro effects of atorvastatin on CD4+ T cells, we compared the mRNA and protein expression of GARP in normal stimulated CD4+ T cells with atorvastatin cocultured stimulated CD4+ T cells. The results of RT-PCR and western blot demonstrated that, compared with normal culture cells, 25 *μ*M atorvastatin coculture significantly upregulated mRNA (^∗^ *P* < 0.01, as in Figure 1(a)) and protein (^∗^ *P* < 0.05, as in Figure 1(b)) expression of GARP in anti-CD3/CD28 stimulated CD4+ T cells. In vivo experiments also indicated that, compared with scrambled group (scrambled), the mRNA (^∗^ *P* < 0.01, as in Figure 1(c)) and protein (^∗^ *P* < 0.05, as in Figure 1(d)) expression of GARP in anti-CD3/CD28 stimulated splenic CD4+ T cells was significantly elevated in oral atorvastatin treatment group (O.A. + scrambled), while GARP-siRNA obviously inhibited the mRNA (both ^∗∗^ *P* and ^∗∗∗^ *P* < 0.05, as in Figure 1(c)) and protein (both ^∗∗^ *P* and ^∗∗∗^ *P* < 0.05, as in Figure 1(d)) expression of GARP in anti-CD3/CD28 stimulated splenic CD4+ T cells in oral atorvastatin + GARP-siRNA group (O.A. + GARP-siRNA). It had been demonstrated that regulatory T cells could secrete many kinds of cytokines (such as IL-10 and TGF-*β*) to suppress the function of T-effector cells. After oral administration of atorvastatin for 4 weeks, the mRNA expression of TGF-*β* in anti-CD3/CD28 stimulated splenic CD4+ T cells were detected in each group. The level of TGF-*β* was significantly upregulated in atorvastatin treated group compared with scrambled group (^∗^ *P* and ^∗∗∗^ *P* < 0.05, as in Figure 1(e)) and GARP-siRNA partly inhibited this elevation of TGF-*β* by atorvastatin (^∗∗^ *P* < 0.05, as in Figure 1(e)). Also, the protein level of TGF-*β* in anti-CD3/CD28 stimulated splenic CD4+ T cells exhibited a same tendency as mRNA in each group (all ^∗^ *P*, ^∗∗^ *P*, and ^∗∗∗^ *P* < 0.05, as in Figure 1(f)). As everyone knows, statin could influence the activity of certain inflammation related signaling pathways such as Smad 2/3 or NF-kb. We detected the change of Smad3 in anti-CD3/CD28 stimulated splenic CD4+ T cells by western blot and indicated significant upregulation of both total Smad3 and phosphorylated Smad3 in oral atorvastatin treatment group (^∗^ *P* < 0.05, as in Figure 1(g)); more interestingly, the knockout of GARP by GARP-siRNA inhibited the upregulation of Smad3 and p-Smad3 induced by atorvastatin (^∗∗^ *P* < 0.05, as in Figure 1(g)).

### 3.2. Atorvastatin Upregulated the Proportion of CD4+LAP+ and CD4+Foxp3+ Tregs in ApoE−/− Splenic Lymphocytes and Enhanced Immunosuppressive Function of Tregs In Vivo; However, Inhibition of GARP Mediated Apoptosis of Tregs

**Figure 2:**
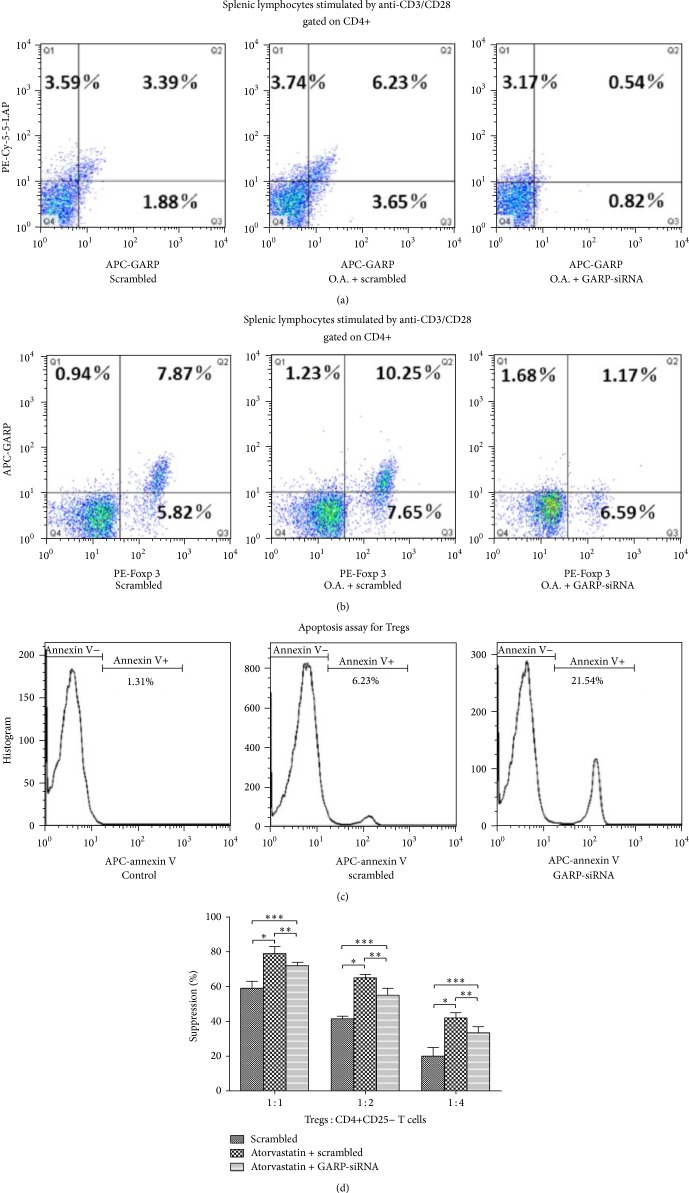
Atorvastatin upregulated the proportion of CD4+LAP+ and CD4+Foxp3+ Tregs in ApoE−/− splenic lymphocytes and enhanced immunosuppressive function of Tregs; however, GARP-siRNA induced apoptosis of Tregs and regulated the immunosuppressive function of Tregs. (a) A percentage of LAP+ and GARP+ cells were determined by surface staining of GARP and LAP after gating on the anti-CD3/CD28 stimulated splenic CD4+ T cells derived from scrambled group (scrambled), oral atorvastatin + scrambled group (O.A. + scrambled), and oral atorvastatin + GARP-siRNA group (O.A. + GARP-siRNA). Data were from 6 mice per group (*n* = 6) and were independently confirmed, all ^∗^ *P*, ^∗∗^ *P*, and ^∗∗∗^ *P* < 0.05. (b) A percentage of Foxp3+ and GARP+ cells were determined by surface staining of GARP and Foxp3 after gating on the anti-CD3/CD28 stimulated splenic CD4+ T cells derived from scrambled group (scrambled), oral atorvastatin + scrambled group (O.A. + scrambled), and oral atorvastatin + GARP-siRNA group (O.A. + GARP-siRNA). Data were from 6 mice per group (*n* = 6) and were independently confirmed, all ^∗^ *P*, ^∗∗^ *P*, and ^∗∗∗^ *P* < 0.05. (c) Apoptosis analysis for scrambled siRNA or GARP-siRNA treated CD4+CD25+ Tregs. The percentage of annexin V+ cells in each groups was showed in histograms, repeated times *n* = 5. (d) Comparisons of Tregs sorted from different treated groups (scrambled, atorvastatin + scrambled or atorvastatin + GARP-siRNA cocultured splenic lymphocytes) against CD4+CD25− T-effector proliferation were shown in bar graphs, repeated times *n* = 5, all ^∗^ *P*, ^∗∗^ *P*, and ^∗∗∗^ *P* < 0.05.

Growing evidences had demonstrated that high dose atorvastatin exhibited a stronger immune regulation than low dose [26]; therefore, we directly adopt high dose oral atorvastatin in ApoE−/− mice. In order to explore the regulation of atorvastatin in Tregs, we detected the proportion of CD4+LAP+ and CD4+Foxp3+ Tregs in anti-CD3/CD28 stimulated CD4+ ApoE−/− splenic lymphocytes by flow cytometry and observed a significant elevation of CD4+LAP+ and CD4+Foxp3+ Tregs in atorvastatin group compared with scrambled group (all ^∗^ *P* < 0.05, as in Figures 2(a) and 2(b)); however, a significant downregulation of CD4+LAP+ and CD4+Foxp3+ Tregs was observed in GARP-siRNA group compared with scrambled and atorvastatin group (all ^∗∗^ *P* and ^∗∗∗^ *P* < 0.05, as in Figures 2(a) and 2(b)). Interestingly, in vitro experimental results indicated that the apoptosis rate of Tregs in GARP-siRNA cocultured lymphocytes was significantly higher than in scrambled and control cocultured lymphocytes (as in Figure 2(c)). In order to further assess the anti-inflammatory effects of Tregs under the influence of atorvastatin and GARP-siRNA, we analyzed the anti-inflammatory function of Tregs sorting from atorvastatin or GARP-siRNA cocultured lymphocytes. At the same Tregs/CD4+CD25− T cells ratio, the anti-inflammatory effects of Tregs on the proliferation of CD4+CD25− T cells were significantly enhanced in the atorvastatin treated group than the scrambled group (all ^∗^ *P* < 0.05, as in Figure 2(d)). However, in the atorvastatin + GARP-siRNA treated group, the anti-inflammatory effects of Tregs were decreased compared to atorvastatin treated group (all ^∗∗^ *P* < 0.05) but still enhanced than scrambled group (all ^∗∗∗^ *P* < 0.05, as in Figure 2(d)). The inhibitory effects of Tregs on CD4+CD25− T cells were concentration dependent (as in Figure 2(d)).

### 3.3. Atorvastatin Increased GARP+ and Foxp3+ Cells, as well as Secretion of Mature TGF-*β* in Atherosclerotic Plaques; However, GARP-siRNA Significantly Suppressed These Functions

**Figure 3:**
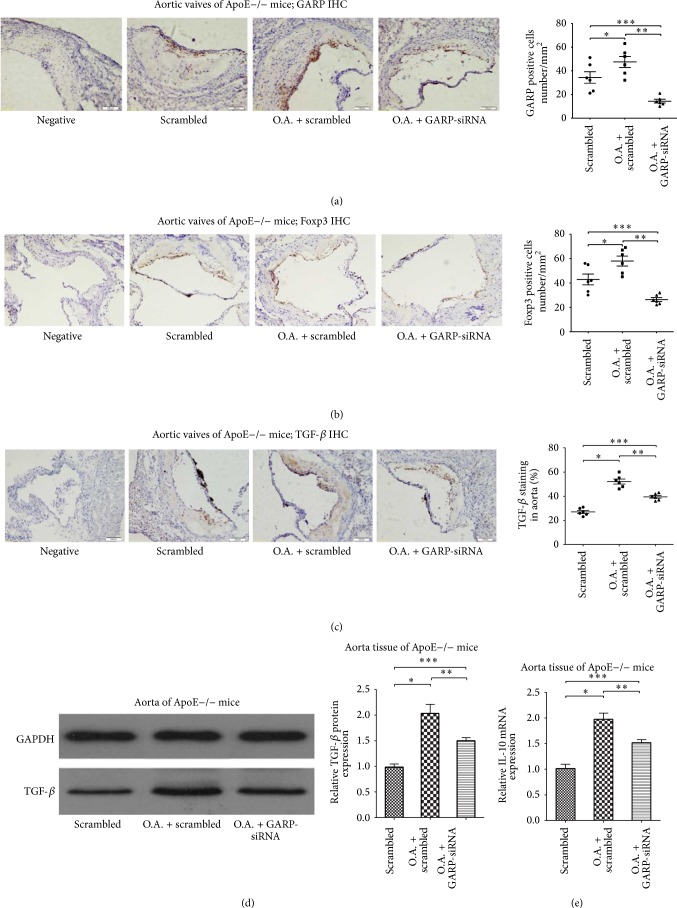
Atorvastatin increased the number of GARP+ and Foxp3+ cells, as well as the secretory mature TGF-*β*1 in atherosclerotic plaques; however, inhibition of GARP by siRNA significantly suppressed these functions: (a) accumulation of GARP+ cells in the atherosclerotic plaques in ApoE−/− mouse aortic root of scrambled group (scrambled), oral atorvastatin + scrambled group (O.A. + scrambled), and oral atorvastatin + GARP-siRNA group (O.A. + GARP-siRNA) was analyzed by immunohistochemistry and shown in scatter diagrams. Data were from 6 mice per group (*n* = 6) and were independently confirmed, all ^∗^ *P*, ^∗∗^ *P*, and ^∗∗∗^ *P* < 0.05. (b) Accumulation of Foxp3+ cells in the atherosclerotic plaques in ApoE−/− mouse aortic root of scrambled group (scrambled), oral atorvastatin + scrambled group (O.A. + scrambled), and oral atorvastatin + GARP-siRNA group (O.A. + GARP-siRNA) was analyzed by immunohistochemistry and shown in scatter diagrams. Data were from 6 mice per group (*n* = 6) and were independently confirmed, all ^∗^ *P*, ^∗∗^ *P*, and ^∗∗∗^ *P* < 0.05. (c) Staining of mature TGF-*β*1 in the atherosclerotic plaques in aortic root of these three groups was analyzed by immunohistochemistry and shown in scatter diagrams. Data were from 6 mice per group (*n* = 6) and were independently confirmed, all ^∗^ *P*, ^∗∗^ *P*, and ^∗∗∗^ *P* < 0.05. (d) The protein expression of mature TGF-*β*1 in aorta of these three groups was analyzed by western blot. Data were from 6 mice per group (*n* = 6) and were independently confirmed, all ^∗^ *P*, ^∗∗^ *P*, and ^∗∗∗^ *P* < 0.05. (e) The relative mRNA expression of IL-10 in aorta of these three groups was analyzed by RT-PCR. Data were from 6 mice per group (*n* = 6) and were independently confirmed, all ^∗^ *P*, ^∗∗^ *P*, and ^∗∗∗^ *P* < 0.05.

After 4 weeks of oral atorvastatin treatment, immunohistochemical assay showed an increase of both GARP+ and Foxp3+ cells, as well as secretory mature TGF-*β* within atherosclerotic plaques (All ^∗^ *P* < 0.05, as in Figures 3(a)~3(c)). Furthermore, the expression of mature TGF-*β* and IL-10 in ApoE−/− mouse aorta was also significantly increased in the atorvastatin group versus the scrambled group (^∗^ *P* < 0.05, Figures 3(d) and 3(e)). However, inhibition of GARP by siRNA obviously decreased the number of both GARP+ and Foxp3+ cells but partly downregulated the secretory mature TGF-*β* within atherosclerotic plaques in atorvastatin + GARP-siRNA group versus atorvastatin treated group (All ^∗∗^ *P* and ^∗∗∗^ *P* < 0.05, as in Figures 3(a)~3(c)). Meanwhile, the expression of mature TGF-*β* and IL-10 in ApoE−/− mouse aorta was also partly reduced in the atorvastatin + GARP-siRNA group versus the atorvastatin group (^∗∗^ *P* and ^∗∗∗^ *P* < 0.05, as in Figures 3(d) and 3(e)).

### 3.4. Atorvastatin Enhanced the Stability of Atherosclerotic Plaques and Improved the Progress of Atherosclerosis in ApoE−/− Mice; However, GARP-siRNA Partly Suppressed These Effects of Atorvastatin

**Figure 4:**
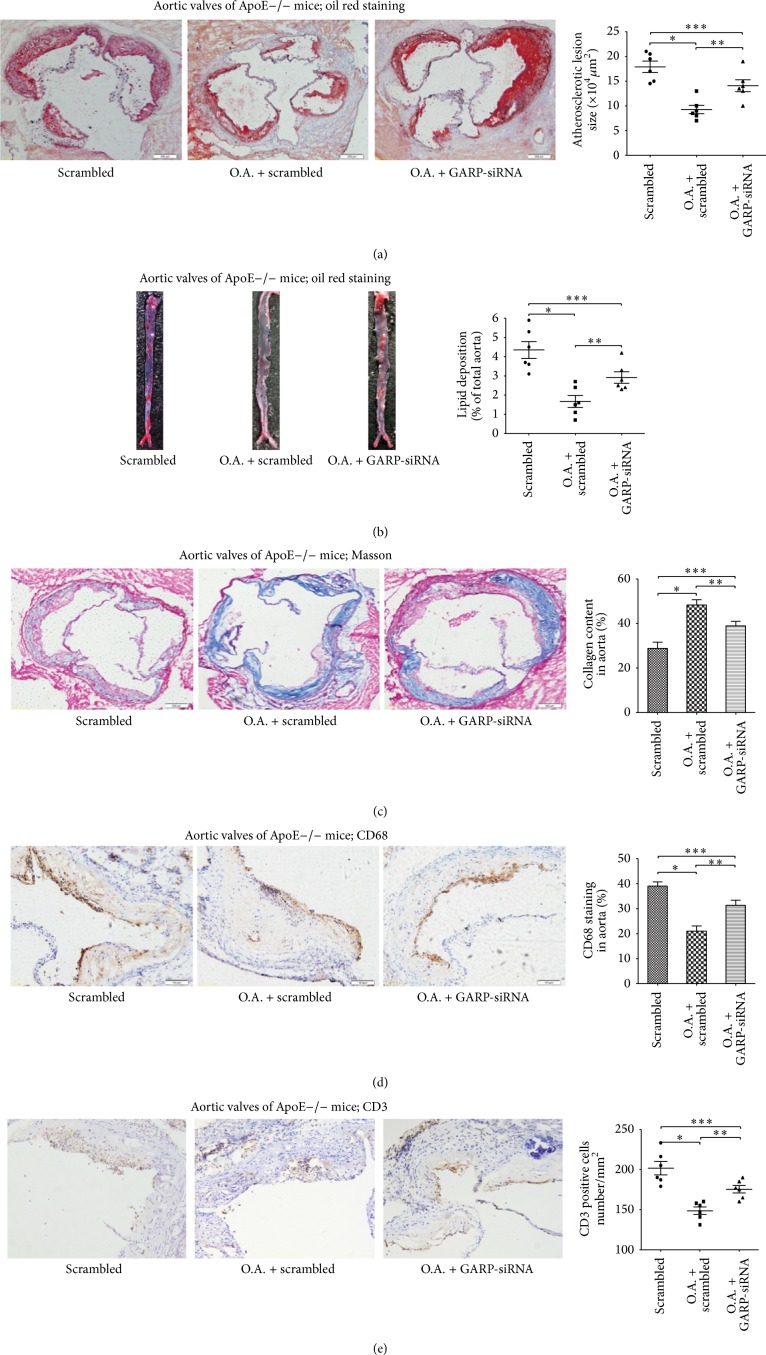
Atorvastatin significantly improved the progression and enhanced the stability of atherosclerotic plaques in ApoE−/− mice, while GARP-siRNA partly inhibited these effects of atorvastatin: (a) oil red O staining and quantitative analysis of atherosclerotic lesion size in ApoE −/− mouse aortic sinus of scrambled group (scrambled), oral atorvastatin + scrambled group (O.A. + scrambled), and oral atorvastatin + GARP-siRNA group (O.A. + GARP-siRNA). Data were from 6 mice per group (*n* = 6) and were independently confirmed, all ^∗^ *P*, ^∗∗^ *P*, and ^∗∗∗^ *P* < 0.05. (b) Oil red O staining and quantitative analysis of atherosclerotic lesion size in ApoE−/− mouse thoracic aortas of these three groups. Data were from 6 mice per group (*n* = 6) and were independently confirmed, all ^∗^ *P*, ^∗∗^ *P*, and ^∗∗∗^ *P* < 0.05. (c) Masson trichrome staining and quantitative analysis of fibrosis in atherosclerotic lesion of aortic sinus in these three groups. Data were from 6 mice per group (*n* = 6) and were independently confirmed, all ^∗^ *P*, ^∗∗^ *P*, and ^∗∗∗^ *P* < 0.05. (d) CD68 staining and quantitative analysis of infiltration of macrophages in atherosclerotic lesion of aortic sinus in these three groups. Data were from 6 mice per group (*n* = 6) and were independently confirmed, all ^∗^ *P*, ^∗∗^ *P*, and ^∗∗∗^ *P* < 0.05. (e) CD3 staining and quantitative analysis of infiltration of T lymphocytes in atherosclerotic lesion of aortic sinus in these three groups. Data were from 6 mice per group (*n* = 6) and were independently confirmed, all ^∗^ *P*, ^∗∗^ *P*, and ^∗∗∗^ *P* < 0.05.

Proximal aortic sections were stained with oil red O to compare the atherosclerotic lesion sizes between the three groups to measure the progress of atherosclerosis. The atherosclerotic lesion sizes were significantly smaller in atorvastatin treated group than scrambled group (^∗^ *P* < 0.05, as in Figure 4(a)), while GARP-siRNA partly inhibited this phenomenon (^∗∗^ *P* and ^∗∗∗^ *P* < 0.05, as in Figure 4(a)). Atherosclerotic lesions were also examined throughout the aorta en face. The percentage of the atherosclerotic lesions to entire aorta was significantly reduced in atorvastatin treated group compared with scrambled group (^∗^ *P* < 0.05, as in Figure 4(b)), while GARP-siRNA partly inhibited this phenomenon (^∗∗^ *P* and ^∗∗∗^ *P* < 0.05, as in Figure 4(b)). The stability of plaques as well as infiltration of macrophages and T lymphocytes in plaques was analyzed by Masson staining and immunohistochemistry of CD68 (for macrophages) and CD3 (for T lymphocytes), respectively. Treatment with atorvastatin significantly enhanced the stability of atherosclerotic plaques (^∗^ *P* < 0.05, as in Figure 4(c)); however, GARP-siRNA partly inhibited the effect of atorvastatin (^∗∗^ *P* and ^∗∗∗^ *P* < 0.05, as in Figure 4(c)). In addition, treatment with atorvastatin significantly decreased the infiltration of macrophages and T lymphocytes in atherosclerotic plaques (^∗^ *P* < 0.05, as in Figures 4(d)~4(e)), while GARP-siRNA partly inhibited this effect of atorvastatin (^∗∗^ *P* and ^∗∗∗^ *P* < 0.05, as in Figures 4(d)~4(e)).

## 4. Discussion

The contribution of inflammation to the progress of atherosclerotic plaque has been an intense area of research [2]. It is well understood that immune determinants such as natural regulatory T cells and immune cytokines control the development of atherosclerosis [7, 27]. Therefore, targeting the transformation of regulatory T cells and immune cytokines in atherosclerotic plaques is important for adequate treatment of atherosclerosis. Numerous studies have declared pleiotropic effects of statins on immune responses and therapeutic value in atherosclerosis [9]. However, the relationship between statin and newly discovered GARP (a potential marker of activated natural regulatory T cells) has not been studied.

We use a siRNA for GARP in this study and performed a sensitive system to characterize this GARP-siRNA. We purify CD4+CD25+ Tregs (Foxp3+ cells >93%) and culture them under the appropriate conditions (anti-CD3/CD28 + IL-2) in vitro which will induce a high expression of GARP/LAP on Tregs. We treat stimulated Tregs with the GARP-siRNA and also pretreat on group of the Tregs with the GARP-siRNA prior to the activation and finally verify the efficiency of the siRNA in downregulating GARP (see Supplement Data A and B in Supplementary Material available online at http://dx.doi.org/10.1155/2015/841472).

Stimulation of T cell receptor (TCR) is needed for the anchoring of GARP on the membrane; hence all our experiments employ the anti-CD3/CD28 stimulated splenic lymphocytes. In in vitro experiments, we observe a significant upregulation of GARP (both mRNA and protein levels) in atorvastatin cocultured stimulated splenic CD4+ T cells (as in Figures 1(a) and 1(b)). Then, we further confirm these results in an ApoE−/− mice model (as in Figures 1(c) and 1(d)). We propose that the upregulation of GARP by atorvastatin is moderate, and certain related downstream pathways (such as the Smad family and Rho family) may be involved in this process. Our results highly indicate Smad3 pathway involved in the biological effects of atorvastatin achieved by upregulating GARP (as in Figure 1(g)).

More recent, researches have indicated that GARP is important for anchoring latent TGF-*β* to the T cells surface as a consequence related to the release of TGF-*β* [17, 23]. Activated regulatory T cells express LAP by the anchoring of GARP, and it is accepted that Tregs exert their anti-inflammatory effects by presenting mature TGF-*β* to effector T cells in a cell-contact dependent manner [28]. Therefore, we also explore the impact of atorvastatin on secretory TGF-*β* of stimulated splenic CD4+ T cells and discover an upregulation of mature TGF-*β* which was parallel to elevation of GARP after oral administration of atorvastatin in ApoE−/− mice (as in Figures 1(e) and 1(f)). This phenomenon highly suggests the close relationship between the anti-inflammatory effects of atorvastatin with GARP/LAP/TGF-*β* compound. It has been reported that GARP could potentiate TGF-*β* signaling in a transgene mouse model [23]. Therefore, we consider that the upregulation of TGF-*β* is highly associated with elevated GARP level in atorvastatin treated CD4+ T cells. Although GARP-siRNA induced a moderate downregulation of TGF-*β*, the administration of atorvastatin still enhanced the expression of TGF-*β* in atorvastatin treated CD4+ T cells (as in Figures 1(e) and 1(f)), indicating that GARP just partly participate in the regulatory effects of atorvastatin on TGF-*β*.

It has been proved in a mouse model that the expression of GARP correlated with the surface LAP, suggesting that the surface LAP is also GARP-anchored in murine T cells [23]. At the same time, TGF-*β* could also induce the surface expression of LAP [20]. As a consequence, we have reasons to believe that atorvastatin could enhance the proportion of CD4+LAP+ regulatory T cells by upregulating the expression of GARP. Our interesting results demonstrate that atorvastatin not only upregulates the mRNA and protein levels of GARP but also enhances the anchoring of GARP and LAP on T cells surface (as in Figure 2(a)), which is the important prerequisite for immune regulation of CD4+LAP+ T cells. Moreover, GARP-siRNA significantly inhibited the proportion of CD4+LAP+ T cells, which indicates that GARP possesses the core effects on the induction of CD4+LAP+ T cells.

It has been well reported that statins could enhance TGF-*β* signal transduction and thereby induce expression of Foxp3 in naïve T cells that were Foxp3− [12]. Our works confirm the upregulation of CD4+Foxp3+ Tregs after administration of atorvastatin (as in Figure 2(b)) and further determine that atorvastatin could directly enhance the expression of GARP in Tregs in vitro (as in Supplement Data A and B). Although, some researchers reported that GARP expression does not control Foxp3 expression and is not involved in induction of Foxp3 expression, they also indicated that some induced Foxp3+ Tregs expressed certain levels of GARP and LAP in mouse, which is markedly different from human iTregs (GARP and LAP negative) [17, 23]. However, some others demonstrated that inhibition of LAP maybe mediate apoptosis of LAP+ Tregs [28]. Our results demonstrate that GARP-siRNA significantly inhibited the proportion of CD4+Foxp3+ regulatory T cells and mediated apoptosis of CD4+Foxp3+ Tregs (as in Figure 2(c)). These results indicate that GARP maybe plays a role in induction of Foxp3+ iTregs by LAP pathway. Moreover, the anti-inflammatory effects of atorvastatin treated Tregs are also regulated by GARP-siRNA (as in Figure 2(d)). The numbers of Tregs sorted from different groups are the same in this Tregs suppression assay; hence, the inhibition of GARP probably disturbs the strengthening of Tregs induced by atorvastatin through certain pathways but does not suppress the essential anti-inflammatory effects of Tregs in control group (as in Figure 2(d)).

It has been reported that the accumulation of TGF-*β*+ cells in atherosclerotic plaques is increased by administration of statin [26, 29]. We observed that atorvastatin increases numbers of GARP+ and Foxp3+ cells and enhances secretion of TGF-*β* in atherosclerotic plaques, while GARP-siRNA significantly reduces the accumulation of GARP+ and Foxp3+ cells but partly inhibits secretion of TGF-*β* in atherosclerotic plaques by atorvastatin (as in Figures 3(a)~3(c)). In addition, the expression of TGF-*β* and IL-10 in atherosclerotic plaques is elevated by administration of atorvastatin and partly decreased by intervention of GARP-siRNA (as in Figures 3(d) and 3(e)). The recruitment of regulatory T cells into inflammatory regions is also regulated by certain signal pathways, and many kinds of chemotactic factors such as CCR family, CX3CR1, and CXCR4 are regarded in the recruitment of Tregs [30]. We do not exclude the possibility of atorvastatin enhancing the recruitment of CD4+LAP+ T cells into inflammatory areas through the GARP/LAP/TGF-*β* pathway.

Active TGF-*β* can be released from the latent TGF-*β*/LTBP complex by the action of *α*V integrins, and it was reported recently that TGF-*β* is released from the latent TGF-*β*/GARP complex through similar mechanisms [22]. The increased regulatory T cells in atherosclerotic plaques by administration of atorvastatin release greater quality of TGF-*β* into inflammatory areas and exert stronger anti-inflammatory effects. The in vivo experiments reveal smaller plaque areas in atorvastatin treated group, but GARP-siRNA partly inhibits this effect of atorvastatin (as in Figures 4(a) and 4(b)). Meanwhile, atorvastatin improved the stability of atherosclerotic plaques and the infiltration of macrophages and effector T cells in atherosclerotic plaques; however, GARP-siRNA partly inhibited these effects of atorvastatin in vivo (as in Figures 4(c)~4(e)). The antiatherosclerosis function of atorvastatin is partly inhibited by GARP-siRNA, which indicates an important role of GARP on anti-inflammatory effect of atorvastatin. Some other mechanisms have been proposed to explain the immunosuppressive effects of the statins including inhibition of antigen presentation by inhibition of the induction of MHC class II expression or blocking of T helper type 1 (Th1) cell differentiation [12]. In addition, it is also reported that the anti-inflammatory effects of statins were mediated through induction of Th2 cells with increased IL-4 production and reduced tumour necrosis factor-*α* and interferon-*γ* production [31]. Statins have also been shown to regulate the production of IL-17 in CD4+ T cells [32]. Our results provide a new evidence that atorvastatin downregulated the percentage of Th1 and Th17 cells and upregulated the percentage of Th2 cells, while GARP-siRNA partly inhibits this effects of atorvastatin (as in Supplement Data C~E), indicating that GARP is also highly associated with the overall state of inflammation in vivo. Certainly, statins are able to act on many kinds of cell types in the immune system [10], and there may be some other cellular targets (such as DCs, endothelial cells, and platelets) involving this process.

## 5. Conclusion

Our works firstly declare the recent discovered CD4+LAP+ regulatory T cells playing an important role in anti-inflammatory effects of atorvastatin, the newly rising activated-Tregs marker GARP being an important status in anti-inflammatory effect of atorvastatin. GARP is a valuable therapeutic target for further research in atherosclerosis.

## Supplementary Material

In supplementary material, we provide evidence about the inhibiting characterization of GARP-siRNA used in this study (see Supplement Data A and B) and the percentage of splenic Th1, Th2 and Th17 cells in different treated groups (see Supplement Data C^~^E).

## Abbreviation

ApoE−/−: Apolipoprotein E gene knockout
TH1: T helper type 1
TH17: T helper type 17
Tregs: Regulatory T cells
IL-10: Interleukin-10
TGF-*β*: Transforming growth factor
HMG-CoA: 3-Hydroxy-3-methylglutaryl coenzyme A
GARP: Glycoprotein A Repetitions Predominant
LAP: TGF-*β* latency-associated protein.
