# Supplementary material for: Atorvastatin Improves Inflammatory Response in Atherosclerosis by Upregulating the Expression of GARP
Source: Mediators Inflamm. 2015 May 10;2015:841472. doi: 10.1155/2015/841472 (PMC4442006; doi:10.1155/2015/841472)

Atorvastatin improve inflammatory response in atherosclerosis by up-regulating the expression of GARP

Authors: Xiaoqi Zhao*; Yuzhou Liu*; Yucheng Zhong*; Bo Liu; Kunwu Yu; Huairui Shi; Ruirui Zhu; Kai Meng; Wei Zhang; Bangwei Wu; Qiutang Zeng#

Authors from the Department of Cardiology, Union Hospital, Tongji Medical College, Huazhong University of Science and Technology.

***These authors contributed equally to this work and should be considered co-first authors.**

#Correspondence to Qiutang Zeng: Qiutang Zeng, MD, Professor, Union Hospital, Tongji Medical College, Huazhong University of Science and Technology, E-mail: zengqiutang@gmail.com, Tel: 008613908640556, fax: 008602787543437

Supplement Data

(A) The relative GARP mRNA expression in scrambled siRNA (MOI=100), GARP-siRNA (MOI=100) or atorvastatin co-cultured activated splenic Tregs and non-activated splenic Tregs, in vitro. Repeated times n=5, and all *p and **p<0.01.

(B) The relative GARP protein expression in scrambled siRNA (MOI=100), GARP-siRNA (MOI=100) or atorvastatin co-cultured activated splenic Tregs, in vitro. Repeated times n=5, and all *p and **p<0.01.

(C) Percentage of Th1 cells were determined by surface staining of CD4 and INFγ in PMA/Ionomycin stimulated splenic lymphocytes derived from scrambled group (Scrambled), oral atorvastatin + scrambled group (O.A. + Scrambled) and oral atorvastatin + GARP-siRNA group (O.A. + GARP-siRNA). Data were from 6 mice per group (n=6) and independently confirmed, all *p, **p and ***p <0.05.

(D) Percentage of Th2 cells were determined by surface staining of CD4 and IL-4 in PMA/Ionomycin stimulated splenic lymphocytes derived from scrambled group (Scrambled), oral atorvastatin + scrambled group (O.A. + Scrambled) and oral atorvastatin + GARP-siRNA group (O.A. + GARP-siRNA). Data were from 6 mice per group (n=6) and independently confirmed, all *p, **p and ***p <0.05.

(E) Percentage of Th17 cells were determined by surface staining of CD4 and IL-17 in PMA/Ionomycin stimulated splenic lymphocytes derived from scrambled group (Scrambled), oral atorvastatin + scrambled group (O.A. + Scrambled) and oral atorvastatin + GARP-siRNA group (O.A. + GARP-siRNA). Data were from 6 mice per group (n=6) and independently confirmed, all *p, **p and ***p <0.05.

Supplement Data:

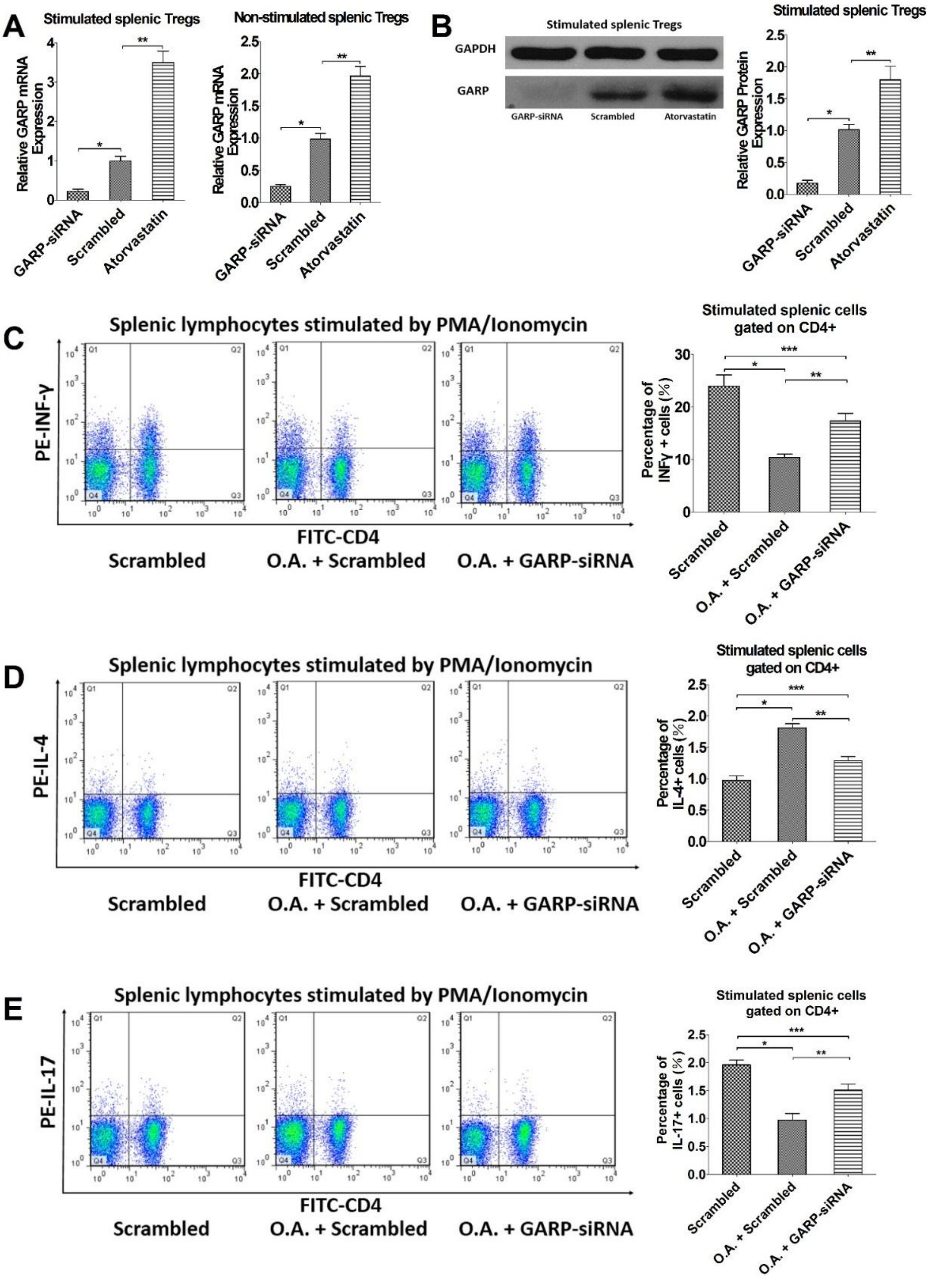

Supplement: Supplementary file 1 — In supplementary material, we provide evidence about the inhibiting characterization of GARP-siRNA used in this study (see Supplement Data A and B) and the percentage of splenic Th1, Th2 and Th17 cells in different treated groups (see Supplement Data C~E). [file 841472.f1.pdf]
